# Dietary Quality and Sociodemographic and Health Behavior Characteristics Among Pregnant Women Participating in the New York University Children's Health and Environment Study

**DOI:** 10.3389/fnut.2021.639425

**Published:** 2021-04-09

**Authors:** Andrea L. Deierlein, Akhgar Ghassabian, Linda G. Kahn, Yelena Afanasyeva, Shilpi S. Mehta-Lee, Sara G. Brubaker, Leonardo Trasande

**Affiliations:** ^1^School of Global Public Health, New York University, New York, NY, United States; ^2^Department of Population Health, New York University Grossman School of Medicine, New York, NY, United States; ^3^Department of Pediatrics, New York University Grossman School of Medicine, New York, NY, United States; ^4^Department of Environmental Medicine, New York University Grossman School of Medicine, New York, NY, United States; ^5^Division of Maternal Fetal Medicine, Department of Obstetrics and Gynecology, New York University Langone Health, New York, NY, United States

**Keywords:** diet, pregnancy, healthy eating index, health behavior, sociodemographic characteristics

## Abstract

Maternal diet, prior to and during pregnancy, plays an important role in the immediate and long-term health of the mother and her offspring. Our objectives were to assess diet quality among a large, diverse, urban cohort of pregnant women, and examine associations with sociodemographic and health behavior characteristics. Data were from 1,325 pregnant women enrolled in New York University Children's Health and Environment Study (NYU CHES). Diet quality was assessed using the Healthy Eating Index (HEI)-2015. Mean total HEI-2015 score was 74.9 (SD = 8.5); 376 (28%), 612 (46%), 263 (20%), and 74 (6%) of women had scores that fell into the grade range of A/B, C, D, and F, respectively. Mean HEI-2015 component scores were high for fruit and whole grains and low for protein-related, sodium, and fat-related components. In multivariable linear regression models, Hispanic women scored 1.65 points higher on the total HEI-2015 (95% CI: 0.21, 3.10) compared to non-Hispanic White women, while younger age (<30 years), parity, single status, pre-pregnancy obesity, smoking, pre-existing hypertension, moderate/severe depressive symptoms, not meeting physical activity recommendations, and not taking a vitamin before pregnancy were associated with ~1.5–5-point lower mean total HEI-2015 scores. Diet is a modifiable behavior; our results suggest a continued need for pre-conceptional and prenatal nutritional counseling.

## Introduction

Maternal diet, both prior to conception and during pregnancy, plays an important role in the immediate and long-term health of the mother and her offspring (1, 2). Poor dietary quality before and during pregnancy is linked to maternal complications, such as gestational diabetes, hypertension, and post-partum depression (3–7). In offspring, *in-utero* exposure to malnutrition, defined as inadequate or excessive nutrient intakes, is associated with a range of adverse short- and long-term health outcomes, including fetal growth restriction, low or high birthweight, and obesity, as well as increased risk of chronic disease in adulthood (1, 8, 9).

It is critical to assess women's dietary quality prior to and during pregnancy with respect to current dietary guidelines and meeting pregnancy-specific recommendations for iron, folate, and other nutrients. Recently, Bodnar et al. described peri-conceptional dietary quality, during the 3 months around conception, of nulliparous women in the United States (U.S.) using the Healthy Eating Index (HEI)-2010 (10). Dietary quality was suboptimal; approximately one-third of women's energy intakes were from empty calories (e.g., added sugars and solid fats), with sugar-sweetened beverages, pasta, and grain desserts ranking among the top energy sources. Lower dietary quality was observed among non-Hispanic Black and Hispanic women, as well as women who did not graduate from college (10). Other studies also report that prenatal dietary quality (using various indices) differs by maternal characteristics and suggest younger age, pre-pregnancy obesity, parity, and smoking are associated with lower scores (2, 11–19). However, there currently remain limitations in the previous literature, including older cohort data collection, homogeneous populations, and lack of consideration of a range of maternal characteristics and health-related factors (2, 11–19).

Given the health implications for the mother and her offspring, there continues to be a need to study women's nutritional status before, during, and after pregnancy, particularly among socio-demographically and geographically diverse populations (19). We can use this information to identify subgroups of women who are at risk of malnutrition, as well as to recognize consumption of specific food groups or nutrients that may be below or exceed recommendations. Our objective in the current study was to assess diet quality using the most recent Dietary Guidelines for Americans 2015–2020 among a contemporary, urban cohort of pregnant women (20). Associations of women's dietary intakes with sociodemographic and health behavior characteristics were examined.

## Materials and Methods

### Study Sample

Data were from the New York University Children's Health and Environment Study (NYU CHES), a prospective birth cohort designed to evaluate the influences of prenatal non-persistent environmental chemical exposures on fetal and post-natal growth and development. The study is described in detail elsewhere (21). Briefly, beginning in 2016, adult women (ages 18 years and older) were enrolled at <18 weeks' gestation from three NYU School of Medicine affiliates in Manhattan and Brooklyn, March 2016–April 2019, post-natal follow-up of these women and their children is ongoing. Women were included if they were pregnant and planned to deliver at one of the study hospitals. All women gave written, informed consent and the study was approved by the institutional review board of the NYU School of Medicine (i15-00778). There were 2,193 women enrolled in NYU CHES; 2,000 women had live births, of which 1,384 completed the dietary assessment. We excluded women with implausible daily energy intakes of <500 kilocalories (*n* = 49) or more than 6,000 kilocalories (*n* = 10). This range was based on plausible intakes for non-pregnant and pregnant women used previously in the literature (22–24). The resulting analytic sample was 1,325 women.

### Dietary Assessment

Women self-administered the electronic version of the Diet History Questionnaire II (DHQ-II, in English and translated to Spanish) to assess their usual dietary intakes during the previous year (25). The DHQ-II has not been validated, but the previous version (DHQ-I) was found to provide reasonable nutrient estimates in adults in validation studies (26). We used the DHQ-II because it captures intakes during the previous 12 months, spanning both prior to and during pregnancy. The DHQ-II is a publicly available food frequency questionnaire developed by the U.S. National Cancer Institute. It consists of 124 commonly consumed food items and includes frequency, portion size, and dietary supplement questions. The median (standard deviation, SD) gestational age of DHQ-II completion was 26 (8.6) weeks: 731 (55%) women completed the DHQ-II in the first/second trimester (<27 weeks' gestation), 512 (39%) women in the third trimester (27 to <40 weeks' gestation), and 82 (6%) women during peri-partum/early post-partum (≥40 weeks' gestation).

### Diet Quality

We assessed diet quality from dietary sources only (excluding vitamin or supplement use) using the HEI-2015 (25). The HEI was developed in 1995 by the U.S. Department of Agriculture and evaluates adherence to national dietary recommendations provided by the Dietary Guidelines for Americans, which are updated every 5 years (25, 27). We selected the HEI-2015 from among several indices available to evaluate diet quality because it reflects the most recent U.S. dietary recommendations for all individuals, the Dietary Guidelines for Americans, 2015–2020 (20). We calculated HEI-2015 scores using resources developed by the National Cancer Institute that are specific to the DHQ-II: the DHQ II Diet^*^Calc software and the HEI-2015 SAS program and required SAS macros (28). Higher HEI scores are associated with favorable infant anthropometric outcomes (e.g., fat mass) (12, 29), as well as reduced risks of chronic diseases and mortality in adults (30).

The HEI-2015 comprises thirteen components (maximum 100 points): nine adequacy components (60 points) and four moderation components (40 points). The nine adequacy components are: total vegetables (5 points), greens and beans (5 points), total fruit (5 points), whole fruit (5 points), whole grains (10 points), total dairy (10 points), total protein foods (5 points), seafood and plant protein (5 points), and fatty acid ratio (ratio of polyunsaturated fats and monounsaturated fats to saturated fat, 10 points). With the exception of fatty acid ratio, all of the adequacy components are scored based on nutrient density per 1,000 calories (e.g., cups of whole fruit per 1,000 calories, see Table 1). The four moderation components are: sodium (10 points), refined grains (10 points), saturated fat (10 points), and added sugars (10 points). Refined grains and sodium are scored based on nutrient density per 1,000 calories; calories from saturated fat and added sugars are scored as a percentage of total energy intake. Higher scores reflect better diet quality; i.e., for the adequacy components, a higher score reflects higher intakes of that component, while for the moderation components, a higher score reflects lower intakes of that component. The thirteen component scores are summed for an overall score (25). A grading approach (A through D and F) has been devised to interpret HEI-2015 scores: A = 90–100; B = 80–89; C = 70–79; D = 60–69; F = 0–59 (25). In NYU CHES, only 23 women had HEI-2015 scores of 90 or greater; therefore, the A and B grade categories were collapsed for statistical analyses. We also examined intakes from dietary sources only of selected nutrients that are needed in increased amounts during pregnancy: vitamin C, iron, folate, vitamin D, vitamin B_1_ (thiamin), vitamin B_2_ (riboflavin), vitamin B_3_ (niacin), vitamin B_6_ (pyroxidine), vitamin B_12_ (cobalamin), selenium, zinc, and docosahexanoic acid (DHA) (31).

**Table 1:** Description of Healthy Eating Index-2015 (HEI-2015) scores and selected nutrient intakes among pregnant women participating in the New York University Children's Health and Environment Study (*n* = 1,325).

	**Score range**	**Standard for maximum scoreStandard for minimum score**	**Mean (SD) score**	**% Meeting maximum score**
Total HEI-2015 score	0–100		74.9 (8.5)	
Adequacy Components				
Total fruits	0–5	≥0.8 cup-equivalents/1,000 kcal No fruit	4.5 (1.0)	71
Whole fruits	0–5	≥0.4 cup-equivalents/1,000 kcal No whole fruit	4.9 (0.5)	92
Total vegetables	0–5	≥1.1 cup-equivalents/1,000 kcal No vegetables	4.3 (1.1)	58
Greens and beans	0–5	≥0.2 cup-equivalents/1,000 kcal No dark green vegetables or beans and peas	4.3 (1.3)	70
Total protein foods	0–5	≥2.5 ounce-equivalents/1,000 kcal No protein foods	3.9 (1.2)	37
Seafood and plant proteins	0–5	≥0.8 cup-equivalents/1,000 kcal No seafood or plant proteins	4.1 (1.3)	61
Whole grains	0–10	≥1.5 ounce-equivalents/1,000 kcal No whole grains	9.7 (1.1)	86
Dairy	0–10	≥1.3 cup-equivalents/1,000 kcal No dairy	6.3 (2.7)	19
Fatty acid ratio	0–10	(PUFAs + MUFAs)/SFAs ≥2.5 (PUFAs + MUFAs)/SFAs ≤1.2	5.4 (3.0)	12
Moderation components				
Refined grains	0–10	≤ 1.8 ounce-equivalents/1,000 kcal ≥4.3 ounce-equivalents/1,000 kcal	8.2 (2.3)	41
Sodium	0–10	≤ 1.1 g/1,000 kcal ≥2.0 g/1,000 kcal	5.3 (2.8)	7
Added sugars	0–10	≤ 6.5% of total kcal ≥26% of total kcal	7.9 (2.8)	31
Saturated fats	0–10	≤ 8% of total kcal ≥16% of total kcal	6.4 (2.9)	16
Selected micronutrients		Pregnancy-specific recommended daily intake	Median (SD) intake	N (%) Meeting recommendations
Vitamin C (milligram, mg)		85	114.3 (125.9)	919 (69)
Iron (mg)		27	12.0 (7.6)	71 (5)
Folate (microgram, mcg daily folate equivalents)		600	487.2 (303.8)	465 (35)
Vitamin D (mcg)		15	3.2 (3.7)	98 (2)
Niacin (mg)		18	15.7 (9.8)	522 (39)
Riboflavin (mg)		1.4	1.8 (1.0)	930 (70)
Thiamin (mg)		1.4	1.2 (0.7)	518 (39)
Vitamin B_6_ (mg)		1.9	1.6 (1.0)	516 (39)
Vitamin B_12_ (mcg)		2.6	3.5 (2.8)	914 (69)
Selenium (mcg)		60	75.1 (47.0)	896 (68)
Zinc (mg)		11	9.0 (5.7)	457 (34)
DHA (mg)		300	50.0 (95.6)	50 (4)

### Sociodemographic and Health Behavior Characteristics

We used electronic health records and questionnaires [administered at <18 weeks' gestation (baseline), at 18–25 weeks' gestation, and after birth] to collect information on sociodemographic characteristics, health behaviors, medical history, mental health, and other factors. In the current analyses, we considered baseline: maternal age (years); highest attained education level (high school graduate or less, some college, college graduate, or graduate/professional degree); household income (< $30,000, $30,000–$99,999, and ≥$100,000); current employment status (yes or no); parity (number of previous births); marital status (single or living with partner/married); smoking status (non-smoker or ever smoking prior to/during pregnancy); consumption of alcoholic beverages (never used, used before pregnancy but not during, used during pregnancy); self-reported pre-existing diabetes (yes or no); self-reported pre-existing hypertension (yes or no); and multi-/prenatal vitamin and/or folic acid supplement use in the month before pregnancy and during pregnancy (vitamin use, yes or no). Self-reported race/ethnicity was categorized as non-Hispanic White, non-Hispanic Black, Hispanic, Asian, and other. Women reporting multiple race (*n* = 33) or other (*n* = 10) were categorized as other. Pre-pregnancy body mass index (BMI, kilograms/meters^2^, kg/m^2^) was calculated using height and self-reported pre-pregnancy weight (from electronic health records and, if missing, from questionnaires) and categorized as underweight (<18.5 kg/m^2^), normal weight (18.5 to <25 kg/m^2^), overweight (25 to <30 kg/m^2^), and obese (≥30 kg/m^2^). There were only 12 underweight women; we included these women with normal weight women for analyses.

The Patient Health Questionnaire-9 (PHQ-9, total points = 27) assessed depressive symptoms and was administered at least once during pregnancy (32). For analyses, we considered only responses from the first administered PHQ-9 [mean (SD) gestational age, 12.7 (5.8) weeks]. We categorized PHQ-9 scores as none/minimal depressive symptoms (0–4 points), mild depressive symptoms (5–9 points), and moderate/severe depressive symptoms (≥10 points) (32). The International Physical Activity Questionnaire-Short Form (IPAQ-SF) queried about physical activity during the previous 7 days (33). The IPAQ-SF was administered up to three times during pregnancy and only responses from the first administration were included in analyses [median (SD) gestational age, 10 (5.5) weeks, 80% within 1st trimester]. Ten women (0.8%) completed the IPAQ after delivering their baby and were instructed to recall their physical activity during their first trimester. We dichotomized women's responses according to meeting the minimal recommended amount of weekly physical activity for pregnant women of at least 150 min of moderate-intensity aerobic activities, including walking (yes or no) (34). We calculated average sleep duration using the difference between usual sleep and wake times based on responses to the following questions, “In the 3 months before you became pregnant, what time did you go to sleep on an average weekday?” and “In the 3 months before you became pregnant, what time did you go to sleep on an average weekday?”. We categorized sleep duration as <7 h, 7 to <9 h, and ≥9 h based on cut-points from previous literature (35). Sleep quality was assessed using the following question, “In the 3 months before you became pregnant, how would you rate the quality of your sleep overall?”, with response choices of very good, fairly good, fairly bad, and very bad. Lastly, we assessed perceived social support using the 5-item ENRICHD Social Support Index (ESSI): “Is there someone available to you whom you can count on to listen to you when you need to talk?”, “Is there someone available to give you good advice about a problem?”, “Is there someone available to you who shows you love and affection?”, “Can you count on anyone to provide you with emotional support (talking over problems or helping you make a difficult decision)?”, and “Do you have as much contact as you would like with someone you feel close to, someone in whom you can trust and confide?” (36, 37). Responses were summed using a point system: 1, none of the time; 2, a little of the time; 3, some of the time; 4, most of the time; and 5, all of the time. Low social support (yes or no) was defined as a response of 3 or lower on at least two of the five items or a total score of 18 or lower (38).

### Statistical Analysis

Data analyses were performed using STATA 15.0 (StataCorp LLC, College Station, TX). Bivariate analyses estimated differences in mean total and component HEI-2015 scores using independent *t*-tests (or F-tests from one-way analysis of variance). We examined differences in total HEI-2015 by timing of DHQ-II completion: 1st/2nd trimester, 3rd trimester, or peri-/post-partum (Supplementary Table 1). Missing data were observed for several maternal characteristics: race/ethnicity (*n* = 2), education (*n* = 12), household income (*n* = 309), insurance (*n* = 9), employment status (*n* = 6), pre-pregnancy BMI (*n* = 10), pre-existing diabetes (*n* = 174), pre-existing hypertension (*n* = 174), depressive symptoms (*n* = 21), sleep duration (*n* = 172), sleep quality (*n* = 179), vitamin use before pregnancy (*n* = 3), vitamin use during pregnancy (*n* = 3), and social support (*n* = 226). Missing data were assumed to be missing at random and were imputed using chained equations (*mi impute chained* command in Stata). We included all maternal characteristics and total HEI scores in the imputation model. During the imputation procedures, pre-pregnancy BMI and depressive symptoms were treated as continuous variables (using predictive mean matching); education and household income were treated as ordinal variables; race/ethnicity, sleep duration, and sleep quality were treated as nominal variables; and employment status, insurance, pre-existing diabetes, pre-existing hypertension, vitamin use, and social support were treated as binary variables. We created 20 imputed data sets. Unadjusted and multivariable linear regression analyses estimated mean differences in total HEI-2015 scores for the maternal characteristics using the imputed data sets (*n* = 1,325). All variables were included in multivariable analyses (Adjusted Model). Regression analyses were repeated using the complete cases for comparison (*n* = 777).

We compared distributions of the selected characteristics of women who were included in analyses (*n* = 1,325) to those who were excluded because of missing or implausible diet data (*n* = 675, Supplementary Table 2). We conducted sensitivity analyses to examine the potential impact for selection bias by estimating inverse probability weights and applying them to regression analyses. We used probit regression with the selected maternal characteristics (Supplementary Table 2; age, race/ethnicity, education, income, marital status, parity, insurance, employment, alcohol use, depressive symptoms, sleep quality, and vitamin use before and during pregnancy) and the resulting predicted probabilities were inverted to determine the final weight (mean 2.00, SD = 1.39). Results from linear regression models with and without weighting did not substantively differ in magnitude or statistical significance. Presented models are unweighted and use multiple imputation.

## Results

Mean (SD) usual energy intake was 1,686 (834) calories, with 51% (10%) of calories derived from carbohydrates, 34% (8%) from fats, and 15% (3%) from proteins. Mean total HEI-2015 score was 74.9 (SD = 8.5, Range = 37.4–94.8); 28, 46, 20, and 6% of women had scores that fell into the grade range of A/B, C, D, and F, respectively (Table 1). Mean HEI-2015 component scores were high for the fruit and whole grains components and low for protein-related, dairy, sodium, added sugars, and fat-related components. Approximately a third of women or fewer met the maximum HEI-2015 component score for total protein foods (37%), dairy (19%), fatty acid ratio (12%), sodium (7%), added sugars (31%), or saturated fats (16%). Mean dietary intakes were above pregnancy-specific recommendations for vitamin C, riboflavin, B_12_, and selenium but below recommendations for iron, vitamin D, and DHA, with 5% or fewer of women meeting recommended intakes for these nutrients (Table 1).

Differences were observed for most of the maternal characteristics between women who were included and excluded from analyses (*p* < 0.05, Supplementary Table 2). Among women included in analyses (*n* = 1,325), more than 80% self-reported as Hispanic (45%) or non-Hispanic White (38%) (Table 2). The majority of women were 30 years or older (67%), under/normal weight (50%), married (90%), currently employed (68%), at least college educated (61%), and nulliparous (52%). Only 11% met physical activity guidelines during pregnancy of 150 min per week. Twelve percent of women reported moderate/severe depressive symptoms during pregnancy and 9% reported sleeping <7 h per night prior to pregnancy. Approximately half of women (51%) reported taking a prenatal vitamin in the month prior to pregnancy, while 87% reported taking a vitamin during pregnancy (Table 2).

**Table 2:** Unadjusted and multivariable linear regression analyses estimating associations of selected maternal characteristics and total Healthy Eating Index-2015 scores among pregnant women in the New York University Children's Health and Environment Study.

**Characteristic**		**Unadjusted^a^**			**Adjusted^b^**		
	**n (%)**	**ß**	**95% CI**	***p***	**ß**	**95% CI**	***p***
Maternal age (years)	1,325						
<25	161 (12)	−4.39	−5.94, −2.84	<0.0001	−5.63	−7.45, −3.81	<0.0001
25 to <30	274 (21)	−2.22	−3.53, −0.91	0.001	−3.20	−4.59, −1.80	<0.0001
30 to <35	513 (39)	0.05	−1.07, 1.16	0.94	−0.68	−1.80, 0.45	0.24
35 or older	377 (28)	Reference			Reference		
Race/Ethnicity	1,323						
Non-Hispanic white	498 (38)	Reference			Reference		
Non-Hispanic black	65 (5)	−0.82	−3.03, 1.38	0.46	1.40	−0.87, 3.67	0.23
Hispanic	591 (45)	−0.48	−1.50, 0.54	0.35	1.65	0.21, 3.10	0.03
Asian	126 (10)	−0.84	−2.50, 0.83	0.33	−0.73	−2.38, 0.91	0.38
Other	43 (3)	−1.77	−4.43, 0.88	0.19	−0.36	−2.93, 2.22	0.79
Highest attained education	1,313						
High school or less	351 (27)	−0.63	−1.84, 0.58	0.31	0.48	−1.42, 2.39	0.62
Some college	160 (12)	−1.04	−2.59, 0.51	0.19	0.96	−0.94, 2.85	0.32
College graduate	386 (29)	−0.58	−1.76, 0.60	0.34	0.04	−1.17, 1.24	0.95
Graduate or professional	416 (32)	Reference			Reference		
Household income	1,016						
< $30,000	203 (20)	−0.54	−1.70, 0.62	0.36	1.92	−0.41, 4.25	0.11
$30,000–99,999	252 (25)	−0.62	−1.83, 0.58	0.31	1.02	−0.53, 2.57	0.20
≥$100,000	561 (55)	Reference			Reference		
Parity	1,325						
Nulliparous	695 (52)	Reference			Reference		
Parous	630 (48)	−0.58	−1.49, 0.34	0.22	−1.55	−2.58, −0.52	0.003
Marital status	1,325						
Married/Living with partner	1,186 (90)	Reference			Reference		
Single	139 (10)	−3.44	−4.93, −1.95	<0.0001	−3.08	−4.64, −1.53	<0.0001
Pre-pregnancy BMI	1,315						
Normal weight	662 (50)	Reference			Reference		
Overweight	377 (29)	0.54	−1.62, 0.53	0.32	−0.64	−1.72, 0.44	0.25
Obese	276 (21)	−1.69	−2.88, −0.50	0.006	−1.42	−2.68, −0.16	0.03
Insurance type	1,316						
Public	635 (48)	−0.45	−1.37, 0.47	0.33	0.67	−0.82, 2.16	0.38
Private	681 (52)	Reference			Reference		
Ever smoked	1,325						
No	1,204 (91)	Reference			Reference		
Yes	121 (9)	−3.88	−5.46, −2.30	<0.0001	−2.39	−3.98, −0.80	0.003
Currently employed	1,319						
No	416 (32)	Reference			Reference		
Yes	903 (68)	−0.27	−1.26, 0.72	0.59	−0.45	−1.59, 0.68	0.44
Pre-existing diabetes	1,151						
No	1,111 (97)	Reference			Reference		
Yes	40 (3)	−1.05	−3.76, 1.65	0.44	−1.21	−3.77, 1.36	0.36
Pre-existing hypertension	1,151						
No	1,103 (96)	Reference			Reference		
Yes	48 (4)	−4.03	−6.69, −1.37	0.003	−3.33	−5.98, −0.69	0.01
Alcohol use	1,325						
Never	399 (30)	Reference			Reference		
Used, stopped during pregnancy	704 (53)	−0.39	−1.44, 0.66	0.46	−0.31	−1.49, 0.86	0.60
Used during pregnancy	222 (17)	−0.71	−2.11, 0.69	0.32	−0.64	−2.16, 0.87	0.41
Depressive symptoms	1,304						
None	726 (56)	Reference			Reference		
Mild	421 (32)	−0.88	−1.90, 0.14	0.09	−0.59	−1.58, 0.40	0.24
Moderate to severe	157 (12)	−3.02	−4.49, −1.56	<0.0001	−1.89	−3.34, −0.43	0.01
Met physical activity guidelines	1,325						
No	1,181 (89)	−2.10	−3.57, −0.63	0.005	−2.10	−3.55, −0.64	0.01
Yes	144 (11)	Reference			Reference		
Average sleep duration (during 3 months before pregnancy)	1,153						
<7 h	122 (9)	−1.83	−3.51, −0.15	0.03	−1.33	−3.04, 0.38	0.13
7 to <9 h	731 (55)	Reference			Reference		
≥9 h	300 (23)	−1.24	−2.36, −0.12	0.03	−0.81	−2.00, 0.38	0.18
Sleep quality (during 3 months before pregnancy)	1,146						
Very good	480 (42)	Reference			Reference		
Fairly good	551 (48)	−1.02	−2.06, 0.01	0.05	−0.72	−1.73, 0.29	0.16
Fairly/Very bad	115 (10)	−2.24	−3.96, −0.53	0.01	−1.10	−2.85, 0.65	0.22
Low social support	1,099						
No	1,002 (91)	−0.68	−2.51, 1.16	0.47	−0.56	−2.41, 1.28	0.55
Yes	97 (9)	Reference			Reference		
Vitamin use before pregnancy	1,322						
No	647 (49)	−2.39	−3.30, −1.48	<0.0001	−1.72	−2.75, −0.69	0.001
Yes	675 (51)	Reference			Reference		
Vitamin use during pregnancy	1,322						
No	176 (13)	−1.16	−2.51, 0.19	0.09	−0.65	−2.07, 0.77	0.36
Yes	1,146 (87)	Reference			Reference		

CI, Confidence interval; BMI, Body mass index

a *Uses data from 20 imputed data sets*.

b *Adjusted model includes all maternal characteristics listed in the table*.

Total HEI-2015 scores varied by sociodemographic characteristics (Table 2, Supplementary Table 3). Younger maternal age (<30 years), single status, pre-pregnancy obesity, any smoking, pre-existing hypertension, reporting moderate to severe depressive symptoms, not meeting physical activity guidelines, self-reported fairly/very bad sleep quality, and not taking a vitamin before pregnancy were associated with lower mean total HEI-2015 scores in unadjusted models. There were no differences in total HEI-2015 and component scores by timing of DHQ-II completion (1st/2nd trimester, 3rd trimester, and peri-/ post-partum), with the exception that women who completed the DHQ-II during pregnancy had slightly higher mean scores for total fruits and sodium, but lower mean scores for total protein foods and saturated fats (Supplementary Table 1).

In multivariable linear regression models (Table 2), Hispanic ethnicity was associated with an ~2-point higher total HEI-2015 score compared to non-Hispanic White (1.65, 95% CI: 0.21, 3.10). Parity, pre-pregnancy obesity, moderate to severe depressive symptoms during pregnancy, not meeting physical activity guidelines, ever smoking, and not using vitamins before pregnancy were each associated with an ~1.5–2.5-point lower total HEI-2015 score. Younger age (<30 years), single status, and pre-existing hypertension were each associated an ~3–5-point lower total HEI-2015 score (Table 2). Results from the regression models restricted to women with complete data were generally of similar direction and magnitude to those using the imputed data.

## Discussion

In this large, diverse, urban cohort of pregnant women, usual dietary intakes within the previous year did not adhere to the most recent recommendations in the Dietary Guidelines for Americans 2015–2020. The average total HEI-2015 score was 74.9, corresponding to a C grade. Although the majority of women achieved high scores for intakes of fruits, vegetables, seafood/plant proteins, and whole grains, the suboptimal total dietary score was reflective of lower scores on components related to dairy, fats, sodium, refined grains, and added sugars. Dietary quality varied by several maternal characteristics. In adjusted models, maternal age <30 years, parity, single status, pre-pregnancy obesity, pre-existing hypertension, ever smoking, moderate/severe depressive symptoms, not meeting physical activity guidelines, and not taking a vitamin prior to pregnancy were associated with lower diet quality scores, with differences ranging from 1.5 to 5 points.

Disparities in dietary quality (assessed using various dietary quality indices) by sociodemographic and lifestyle characteristics are commonly reported among pregnant populations (2, 10–15, 17–19, 39, 40). Consistent with findings from the current study, dietary quality is often lower among women who are low-income, less-educated, or smokers (10–15, 39), while dietary quality is higher among women who are older or nulliparous (13, 15). Maternal obesity is also associated with poor diet quality (12, 14, 17, 18, 39, 40) and there is some evidence that diet quality among women with obesity may worsen throughout pregnancy and the post-partum period (40). In the current study, women with pre-pregnancy obesity had lower total HEI-2015 scores. This is of concern because maternal obesity is an independent risk factor for micronutrient deficiencies (e.g., iron and folate), as well as pregnancy complications and adverse health outcomes, such as gestational diabetes, birth defects, macrosomia, and childhood obesity (8, 9, 41). Nutritional counseling may help to at least partially alleviate many of these outcomes by improving women's dietary intakes prior to and during pregnancy (42). Additionally, we found that pre-existing hypertension, but not diabetes, was associated with lower HEI-2015 scores. Lower dietary quality is often linked with increased risk of these conditions (43–45). Women with diabetes prior to pregnancy may be more likely to modify their diets as part of a treatment program, compared to women with hypertension, who may be more reliant on blood pressure lowering medications to control their condition.

Evidence supporting maternal race/ethnicity as an independent predictor of diet quality is inconsistent among non-White race/ethnic groups (10, 12, 13, 15, 17, 39), but studies often lack adequately diverse populations needed to make comparisons (11, 14, 18). Among a large, multi-site pregnancy cohort, Bodnar et.al. found that non-Hispanic Black and Hispanic women had lower peri-conceptional HEI-2010 scores than non-Hispanic White women, which was attributed to greater consumption of sugar-sweetened beverages and lower consumption of nutrient-dense foods, such as fruits, vegetables, whole grains, and dairy products (10). Other studies, among participants of a large birth cohort in North Carolina (using the Diet Quality Index for Pregnancy) and among participants of the National Health and Nutrition Examination Surveys (2003–2012, using the HEI-2010), reported similar or better diet quality among non-White women compared to White women (15, 17, 39). In the current study, mean total HEI-2015 score was nearly 2 points higher among Hispanic women compared to non-Hispanic White women in adjusted models, with no differences observed for non-Hispanic Black, Asian, or other race/ethnic groups. Discrepancies in findings between studies likely highlight underlying differences in study populations related to maternal social, behavioral, environmental, or cultural characteristics (e.g., urban vs. rural settings or level of acculturation), which are strongly associated with race/ethnicity and not always captured in statistical analyses (13).

In addition to smoking, we identified two other health behaviors that were associated with lower diet quality scores: not meeting minimum physical activity recommendations and not taking a multivitamin or supplement before pregnancy. Only 11% of women met the minimum recommendations for physical activity during pregnancy of 150 min per week, equivalent to brisk walking for 30 min on most days of the week. A growing body of evidence demonstrates that exercise during pregnancy is safe and provides numerous health benefits for both mother and baby, including lower risks of pregnancy complications, poor birth outcomes, maternal weight retention, and post-partum depression (34). Exercise may encourage healthy eating and is positively related to nutrient intakes in pregnant (18) and non-pregnant populations (46), which is consistent with our findings in pregnant women and suggests that exercise is an important target for interventions. Similar to physical activity, multivitamin use before pregnancy may also serve as an indicator of women's overall health behaviors and be used to identify women who are at risk for inadequate nutrient stores and intakes during pregnancy (39). In our study, only half of women reported taking a vitamin or supplement before pregnancy. This is concerning given that mean dietary intakes of iron, folate, vitamin D, and DHA, which are critical for optimal fetal development, were below pregnancy-specific recommendations, with only one third of women meeting recommendations for folate (47). Recent national data show that multivitamin use remains low among women ages 18–44 years; ~38 and 21% of women who report trying to get pregnant and not trying to get pregnant, respectively, take a daily multivitamin (48). Contrary to studies in non-pregnant populations, sleep duration and sleep quality were not associated with diet quality in our study (35). In unadjusted models, women with short (<7 h) and long (≥9 h) weekday sleep durations had lower HEI-2015 scores, but these associations were attenuated in multivariable models. Few studies have examined this association among women around the time of pregnancy; however, studies among pregnant women (49, 50) and mothers (51) suggest a relationship between poor sleep characteristics and unhealthy dietary intakes. Pre-conceptional messaging from health care providers and public health practitioners regarding these modifiable health behaviors is necessary to encourage women to improve their nutritional and overall health status, especially among those who are considering pregnancy.

We also found that reporting moderate to severe depressive symptoms during pregnancy was associated with lower dietary quality. Our findings for depressive symptoms are consistent with previous, though limited, literature suggesting that maternal mental health, including depression and anxiety, is associated with unhealthy dietary intakes (using various dietary assessments) (52, 53). This is of concern because both poor maternal mental health and poor dietary intakes increase the risk of adverse birth outcomes, such as preterm birth and low birth weight (54, 55), and later childhood health outcomes (56, 57). In non-pregnant populations, consumption of healthy foods with low dietary glycemic index/load, particularly those containing omega-3 fatty acids (e.g., DHA) and antioxidants, may prevent or lessen depressive symptoms (58, 59). Although evidence during pregnancy is limited, women with a history of depression or reporting depressive symptoms should be a priority for prenatal nutrition counseling given the benefits for mother and baby (60). Lastly, we did not observe an association between social support (which excluded spousal/partner support) and diet quality. Results from previous studies of pregnant women are inconsistent (18, 61, 62) but there is notable variability in their assessment methods, statistical analyses, and study population characteristics, which may account for discrepant findings. Social support is likely inter-related with socioeconomic and cultural factors. More research is needed to understand how social support may influence diet and other health behaviors among high-risk groups of women, such as those who are single or living in low resource settings.

Main strengths of this study include the large, urban population of pregnant women that was heterogeneous across a range of maternal sociodemographic and health behavior characteristics, as well as the use of the most recent dietary recommendations. Limitations of this study include our dietary assessment method. The DHQ-II assessed dietary intakes for the previous year, capturing usual intakes prior to and during pregnancy for the majority of women, which precluded our ability to distinguish specific timeframes for women's dietary intakes. It is not clear how dietary assessments completed during pregnancy may influence the recall of intakes during a time period that spans both preconception and pregnancy; to our knowledge, there are no studies that examine the validity of a 12-month dietary assessment tool self-administered during pregnancy. There is little evidence that women greatly alter or improve their dietary intakes during pregnancy (63–66), with the exception of increases in fruit intakes (64, 66). We compared diet quality scores by timing of DHQ-II completion and observed no differences in total HEI-2015 score and the majority of component scores among women who completed the DHQ-II during the peri-/post-partum period compared to those who completed it during pregnancy. Demographic characteristics of women with live deliveries enrolled in NYU CHES were very similar to those of pregnant women in New York City (21); however, we did observe differences in characteristics of women who did and did not complete the DHQ-II. Although there were no substantive differences in our results after weighting them, we cannot exclude the possibility that our results may be biased and have limited generalizability. Several of the maternal characteristics, including younger age and single status, were associated with lower diet quality, potentially underestimating differences in dietary quality based on these variables. It should also be noted that the IPAQ-SF only queried physical activities during the previous week and has limited validity compared to objective measures of physical activity, particularly among pregnant women (67). We dichotomized reported weekly duration of physical activities according to current recommendations, but misclassification is still possible, which would underestimate our results.

## Conclusion

Consistent with previous literature, evidence from the current study suggests that sub-optimal dietary quality is common among women prior to and during pregnancy and that several maternal characteristics, such as young maternal age, parity, smoking, not taking a vitamin before pregnancy, and reporting depressive symptoms, may be associated with lower diet quality scores. Pregnancy (and lactation) substantially increase nutritional demands to an extent that may compromise even women who enter pregnancy well-nourished. This is of clinical and public health concern because poor nutrient intakes during pregnancy are associated with a range of adverse health outcomes in both the mother and her child. Diet represents a modifiable behavior and continued emphasis should be placed on the provision of pre-conceptional and prenatal nutritional counseling, particularly among high-risk women who are in need of dietary intervention.

## Data Availability Statement

The datasets generated for this article are not readily available because data may be shared pursuant to ECHO program regulations. Requests to access the datasets should be directed to LT, leonardo.trasande@nyulangone.org.

## Ethics Statement

The studies involving human participants were reviewed and approved by institutional review board of the NYU School of Medicine (i15-00778). The patients/participants provided their written informed consent to participate in this study.

## Author Contributions

AD conducted the secondary analyses and wrote the first draft of the manuscript. AG, LK, YA, SM-L, SB, and LT contributed to the design of the study and acquisition of the data. All authors contributed revisions to the manuscript and approved the final manuscript for submission.

## Conflict of Interest

The authors declare that the research was conducted in the absence of any commercial or financial relationships that could be construed as a potential conflict of interest.
