# Supplementary material for: Dietary Quality and Sociodemographic and Health Behavior Characteristics Among Pregnant Women Participating in the New York University Children's Health and Environment Study
Source: Front Nutr. 2021 Apr 9;8:639425. doi: 10.3389/fnut.2021.639425 (PMC8062781; doi:10.3389/fnut.2021.639425)
Supplement: Supplementary file 1 [file Table_1.DOCX]

| Supplemental Table 1. Mean (SD) total and component HEI-2015 scores by timing of Diet History Questionnaire (DHQ)-II completion, 1^st^/2^nd^ trimester (<27 weeks’ gestation), 3^rd^ trimester (27 to <40 weeks’ gestation), and peri-/postpartum (40 weeks’ gestation or later) among pregnant women participating in the New York University Children’s Health and Environment Study (n=1,325). | | | | |
| --- | --- | --- | --- | --- |
| Dietary Variable | Timing of DHQ-II Completion | | |  |
|  | 1^st^/2^nd^ Trimester  (n=731) | 3^rd^ Trimester (n=512) | Peri-/Postpartum (n=82) | p |
| Total HEI Score | 74.5 (8.4) | 75.5 (8.8) | 75.4 (7.7) | 0.10 |
| Total Fruits | 4.4 (1.1) | 4.6 (0.9) | 4.3 (1.2) | 0.001 |
| Whole Fruits | 4.9 (0.6) | 4.9 (0.5) | 4.8 (0.6) | 0.08 |
| Total Vegetables | 4.3 (1.1) | 4.3 (1.1) | 4.5 (1.0) | 0.39 |
| Greens and Beans | 4.3 (1.3) | 4.3 (1.4) | 4.3 (1.3) | 0.81 |
| Total Protein Foods | 3.9 (1.2) | 3.8 (1.3) | 4.1 (1.2) | 0.01 |
| Seafood and Plant Proteins | 4.2 (1.3) | 4.1 (1.4) | 4.2 (1.3) | 0.12 |
| Whole Grains | 9.7 (1.1) | 9.6 (1.2) | 9.5 (1.3) | 0.17 |
| Dairy | 6.2 (2.7) | 6.4 (2.8) | 6.3 (2.7) | 0.25 |
| Fatty Acids | 5.4 (2.9) | 5.3 (3.1) | 5.6 (2.9) | 0.78 |
| Refined Grains | 8.1 (2.3) | 8.2 (2.3) | 8.1 (2.4) | 0.62 |
| Sodium | 5.1 (2.8) | 5.7 (2.8) | 4.9 (2.8) | 0.0004 |
| Added Sugars | 8.0 (2.8) | 7.7 (3.0) | 8.0 (2.6) | 0.15 |
| Saturated Fats | 6.1 (2.9) | 6.7 (2.9) | 6.8 (2.8) | 0.001 |
