# Supplementary material for: Dietary Quality and Sociodemographic and Health Behavior Characteristics Among Pregnant Women Participating in the New York University Children's Health and Environment Study
Source: Front Nutr. 2021 Apr 9;8:639425. doi: 10.3389/fnut.2021.639425 (PMC8062781; doi:10.3389/fnut.2021.639425)
Supplement: Supplementary file 2 [file Table_2.DOCX]

| Supplementary Table 2. Baseline characteristics of women participating in the New York University Children’s Health and Environment Study who were included in (n=1,325) or excluded from (n=675) the analysis because of missing data on diet. | | | |
| --- | --- | --- | --- |
| Characteristic | Included | Excluded | p^a^ |
|  | n (%) | n (%) |  |
| Maternal Age (years) | 1325 | 675 | 0.04 |
| <25 | 161 (12) | 101 (15) |  |
| 25 to <30 | 274 (21) | 151 (22) |  |
| 30 to <35 | 513 (39) | 219 (32) |  |
| 35 or older | 377 (28) | 204 (30) |  |
|  |  |  |  |
| Race/Ethnicity | 1323 | 672 | <0.0001 |
| Non-Hispanic White | 498 (38) | 179 (27) |  |
| Non-Hispanic Black | 65 (5) | 49 (7) |  |
| Hispanic | 591 (45) | 374 (56) |  |
| Asian | 126 (9) | 54 (8) |  |
| Other | 43 (3) | 16 (2) |  |
|  |  |  |  |
| Highest Attained Education | 1313 | 670 | <0.0001 |
| High School Graduate or Less | 351 (27) | 284 (42) |  |
| Some College | 160 (12) | 69 (10) |  |
| College Graduate | 386 (29) | 169 (25) |  |
| Graduate or Professional School | 416 (32) | 148 (22) |  |
|  |  |  |  |
| Household Income | 1016 | 456 | <0.0001 |
| <$30,000 | 203 (20) | 117 (26) |  |
| $30,000 – 99,999 | 252 (25) | 141 (31) |  |
| >=$100,000 | 561 (55) | 198 (43) |  |
|  |  |  |  |
| Marital Status | 1325 | 674 | 0.002 |
| Married/Living with Partner | 1186 (90) | 571 (85) |  |
| Single | 139 (10) | 103 (15) |  |
|  |  |  |  |
| Parity | 1325 | 672 | 0.004 |
| Nulliparous | 695 (52) | 307 (46) |  |
| Parous | 630 (48) | 365 (54) |  |
|  |  |  |  |
| Pre-Pregnancy BMI | 1315 | 666 | 0.83 |
| Normal Weight | 662 (50) | 326 (49) |  |
| Overweight | 377 (29) | 194 (29) |  |
| Obese | 276 (21) | 146 (22) |  |
|  |  |  |  |
| Insurance Type | 1316 | 667 | <0.0001 |
| Public | 635 (48) | 384 (58) |  |
| Private | 681 (52) | 283 (42) |  |
|  |  |  |  |
| Currently Employed | 1319 | 673 | <0.0001 |
| No | 416 (32) | 270 (40) |  |
| Yes | 903 (68) | 403 (60) |  |
|  |  |  |  |
| Ever Smoked | 1325 | 672 | 0.78 |
| No | 1204 (91) | 608 (90) |  |
| Yes | 121 (9) | 64 (10) |  |
|  |  |  |  |
| Pre-existing Diabetes | 1151 | 500 | 0.94 |
| No | 1111 (97) | 483 (97) |  |
| Yes | 40 (3) | 17 (3) |  |
|  |  |  |  |
| Pre-existing Hypertension | 1151 | 499 | 0.83 |
| No | 1103 (96) | 477 (96) |  |
| Yes | 48 (4) | 22 (4) |  |
|  |  |  |  |
| Alcohol Use | 1325 | 669 | <0.0001 |
| Never | 399 (30) | 263 (39) |  |
| Used but stopped during pregnancy | 704 (53) | 320 (48) |  |
| Used during pregnancy | 222 (17) | 86 (13) |  |
|  |  |  |  |
| Depressive Disorders | 1304 | 608 | 0.03 |
| None | 726 (56) | 307 (50) |  |
| Mild | 421 (32) | 203 (33) |  |
| Moderate to Severe | 157 (12) | 98 (16) |  |
|  |  |  |  |
| Met Physical Activity Guidelines | 1325 | 654 | 0.001 |
| No | 1181 (89) | 613 (94) |  |
| Yes | 144 (11) | 41 (6) |  |
|  |  |  |  |
| Sleep Duration | 1153 | 500 | 0.001 |
| <7 hours | 122 (11) | 51 (10) |  |
| 7 – <9 hours | 731 (63) | 273 (55) |  |
| >=9 hours | 300 (26) | 176 (35) |  |
| Sleep Quality | 1146 | 496 | 0.60 |
| Very Good | 480 (42) | 221 (45) |  |
| Fairly Good | 551 (48) | 228 (46) |  |
| Fairly/Very Bad | 115 (10) | 47 (9) |  |
|  |  |  |  |
| Low Social Support | 1099 | 311 | 0.07 |
| No | 1002 (91) | 273 (88) |  |
| Yes | 97 (9) | 38 (12) |  |
|  |  |  |  |
| Vitamin Use Before Pregnancy | 1322 | 661 | <0.0001 |
| No | 647 (49) | 394 (60) |  |
| Yes | 675 (51) | 267 (40) |  |
|  |  |  |  |
| Vitamin Use During Pregnancy | 1322 | 663 | 0.01 |
| No | 176 (13) | 119 (18) |  |
| Yes | 1146 (87) | 544 (82) |  |
| Body mass index, BMI  ^a^p-value from χ^2^ test |  |  |  |
