# Supplementary material for: Dietary Quality and Sociodemographic and Health Behavior Characteristics Among Pregnant Women Participating in the New York University Children's Health and Environment Study
Source: Front Nutr. 2021 Apr 9;8:639425. doi: 10.3389/fnut.2021.639425 (PMC8062781; doi:10.3389/fnut.2021.639425)
Supplement: Supplementary file 3 [file Table_3.DOCX]

| Supplemental Table 3. Bivariate analyses of mean total Health Eating Index 2015 (HEI-2015) scores with selected characteristics of pregnant women participating in the New York University Children’s Health and Environment Study. | | | |  |
| --- | --- | --- | --- | --- |
| Characteristic | n (%) | Total HEI-2015 Score, Mean (SD) | p^a^ | |
|  |  |  |  |  |
| Maternal Age (years) | 1325 |  |  | |
| <25 | 161 (12) | 71.5 (8.9) |  | |
| 25 to <30 | 274 (21) | 73.7 (9.0) | <0.0001 | |
| 30 to <35 | 513 (39) | 76.0 (8.1) |  | |
| 35 or older | 377 (28) | 75.9 (8.0) |  | |
|  |  |  |  | |
| Race/Ethnicity | 1323 |  |  | |
| Non-Hispanic White | 498 (38) | 75.3 (8.2) |  | |
| Non-Hispanic Black | 65 (5) | 74.5 (8.6) |  | |
| Hispanic | 591 (45) | 74.9 (8.9) | 0.60 | |
| Asian | 126 (10) | 74.5 (8.6) |  | |
| Other | 43 (3) | 73.5 (7.3) |  | |
|  |  |  |  | |
| Highest Education Level | 1313 |  |  | |
| High School or Less | 351 (27) | 74.8 (9.1) |  | |
| Some College | 160 (12) | 74.4 (9.1) | 0.59 | |
| College | 386 (29) | 74.8 (8.3) |  | |
| Graduate/Professional | 416 (32) | 75.4 (8.0) |  | |
|  |  |  |  | |
| Household Income | 1016 |  |  | |
| <$30,000 | 203 (20) | 74.1 (9.1) |  | |
| $30,000 – 99,999 | 252 (25) | 73.9 (9.1) | 0.06 | |
| >=$100,000 | 561 (55) | 75.3 (7.9) |  | |
|  |  |  |  | |
| Parity | 1325 |  |  | |
| Nulliparous | 695 (52) | 75.2 (8.3) | 0.22 | |
| Parous | 630 (48) | 74.6 (8.7) |  | |
|  |  |  |  | |
| Marital Status | 1325 |  |  | |
| Married/Living with Partner | 1186 (90) | 75.3 (8.1) | <0.0001 | |
| Single | 139 (10) | 71.9 (10.5) |  | |
|  |  |  |  | |
| Pre-Pregnancy BMI | 1315 |  |  | |
| Normal Weight | 662 (50) | 75.5 (8.2) |  | |
| Overweight | 377 (29) | 74.9 (8.8) | 0.02 | |
| Obese | 276 (21) | 73.8 (8.9) |  | |
|  |  |  |  | |
| Insurance Type | 1316 |  |  | |
| Public | 635 (48) | 74.7 (8.9) | 0.30 | |
| Private | 681 (52) | 75.2 (8.2) |  | |
|  |  |  |  | |
| Ever Smoked | 1325 |  |  | |
| No | 1204 (91) | 75.3 (8.3) | <0.0001 | |
| Yes | 121 (9) | 71.4 (9.4) |  | |
|  |  |  |  | |
| Currently Employed | 1319 |  |  | |
| No | 416 (32) | 75.1 (9.3) | 0.57 | |
| Yes | 903 (68) | 74.9 (8.2) |  | |
|  |  |  |  | |
| Pre-existing Diabetes | 1151 |  |  | |
| No | 1111 (97) | 75.0 (8.4) | 0.56 | |
| Yes | 40 (3) | 74.2 (9.5) |  | |
|  |  |  |  | |
| Pre-existing Hypertension | 1151 |  |  | |
| No | 1103 (96) | 75.2 (8.4) | 0.002 | |
| Yes | 48 (4) | 71.3 (9.5) |  | |
|  |  |  |  | |
| Alcohol Use | 1325 |  |  | |
| Never used | 399 (30) | 75.3 (8.6) |  | |
| Used but stopped during pregnancy | 704 (53) | 74.9 (8.7) | 0.59 | |
| Used during pregnancy | 222 (17) | 74.6 (7.8) |  | |
|  |  |  |  | |
| Depressive Symptoms | 1304 |  |  | |
| None | 726 (56) | 75.6 (8.1) |  | |
| Mild | 421 (32) | 74.7 (8.5) | 0.0002 | |
| Moderate to Severe | 157 (12) | 72.6 (9.7) |  | |
|  |  |  |  | |
| Met Physical Activity Guidelines | 1325 |  |  | |
| No | 1181 (89) | 74.7 (8.5) | 0.005 | |
| Yes | 144 (11) | 76.8 (8.3) |  | |
|  |  |  |  | |
| Average Sleep Duration (during 3 months before pregnancy) | 1153 |  |  | |
| <7 hours | 249 (22) | 73.5 (9.5) |  | |
| 7 – <9 hours | 438 (38) | 75.6 (8.0) | 0.005 | |
| >= 9 hours | 320 (28) | 74.2 (8.9) |  | |
|  |  |  |  | |
| Sleep Quality (during 3 months before pregnancy) | 1146 |  |  | |
| Very Good | 480 (42) | 75.7 (8.5) |  | |
| Fairly Good | 551 (48) | 74.7 (8.3) | 0.03 | |
| Fairly/Very Bad | 115 (10) | 73.6 (8.9) |  | |
|  |  |  |  | |
| Low Social Support | 1099 |  |  | |
| No | 1002 (91) | 75.0 (8.5) | 0.52 | |
| Yes | 97 (9) | 74.5 (8.0) |  | |
|  |  |  |  | |
| Vitamin Use Before Pregnancy | 1322 |  |  | |
| No | 647 (49) | 73.7 (8.9) | <0.0001 | |
| Yes | 675 (51) | 76.1 (8.0) |  | |
|  |  |  |  | |
| Vitamin Use During Pregnancy | 1322 |  |  | |
| No | 176 (13) | 73.9 (8.4) | 0.10 | |
| Yes | 1146 (87) | 75.1 (8.5) |  | |

Standard deviation, SD; body mass index, BMI

^a^p-value from *t*-test (dichotomous variables) or F test (categorical variables); ^b^p-value from χ^2^ test
